# The N-Terminal Intrinsically Disordered Domain of Mgm101p Is Localized to the Mitochondrial Nucleoid

**DOI:** 10.1371/journal.pone.0056465

**Published:** 2013-02-13

**Authors:** David C. Hayward, Zsuzsanna Dosztányi, George Desmond Clark-Walker

**Affiliations:** 1 Evolution, Ecology and Genetics, Research School of Biology, Australian National University, Canberra, Australian Capital Territory, Australia; 2 Institute of Enzymology, Research Centre for Natural Sciences, Hungarian Academy of Sciences, Budapest, Hungary; 3 Biological Chemistry, Research School of Chemistry, Australian National University, Canberra, Australian Capital Territory, Australia; University of South Florida College of Medicine, United States of America

## Abstract

The mitochondrial genome maintenance gene, *MGM101*, is essential for yeasts that depend on mitochondrial DNA replication. Previously, in *Saccharomyces cerevisiae*, it has been found that the carboxy-terminal two-thirds of Mgm101p has a functional core. Furthermore, there is a high level of amino acid sequence conservation in this region from widely diverse species. By contrast, the amino-terminal region, that is also essential for function, does not have recognizable conservation. Using a bioinformatic approach we find that the functional core from yeast and a corresponding region of Mgm101p from the coral *Acropora millepora* have an ordered structure, while the N-terminal domains of sequences from yeast and coral are predicted to be disordered. To examine whether ordered and disordered domains of Mgm101p have specific or general functions we made chimeric proteins from yeast and coral by swapping the two regions. We find, by an *in vivo* assay in *S.cerevisiae*, that the ordered domain of *A.millepora* can functionally replace the yeast core region but the disordered domain of the coral protein cannot substitute for its yeast counterpart. Mgm101p is found in the mitochondrial nucleoid along with enzymes and proteins involved in mtDNA replication. By attaching green fluorescent protein to the N-terminal disordered domain of yeast Mgm101p we find that GFP is still directed to the mitochondrial nucleoid where full-length Mgm101p-GFP is targeted.

## Introduction

A distinctive feature of respiring eukaryotic cells is the mitochondrion. This organelle is the site for electron transport, oxygen consumption and ATP synthesis collectively termed oxidative phosphorylation. In most eukaryotes some components of oxidative phosphorylation are encoded by the organelle's genome that varies in size and may have from 3 to 67 protein-coding genes [1]. However, a large majority of mitochondrial proteins are coded by nuclear DNA, made in the cytoplasm and imported. Proteins required for mitochondrial DNA (mtDNA) replication, repair, distribution, packaging and transcription are all imported. Such proteins, together with mtDNA, are located in nucleoids named by analogy to similar bodies in bacteria. Nucleoids are attached to the inner membrane on the matrix side and have been shown in yeasts and mammals to contain over 20 proteins, some of which do not have recognizable roles in mtDNA transactions [2]–[6]. Mitochondrial nucleoids in mammals are thought to have a layered structure where components involved in mtDNA replication and transcription occupy a central core while other proteins are located in the periphery [2]. Such an organization appears to preclude mixing of mtDNA between nucleoids [7]. Notable components of nucleoids in yeasts are mtDNA polymerase, Mip1, single stranded binding protein, Rim1, a mtDNA packaging protein, Abf2, a protein for transcription, Rpo41 and a protein for mitochondrial genome maintenance, Mgm101.

In *Saccharomyces cerevisiae* the mitochondrial genome maintenance gene, *MGM101*, encodes a protein of 269 amino acids [8]. The mature polypeptide has 247 amino acids after cleavage of an amino-terminal 22 amino acid mitochondrial targeting signal [9]. This gene is vital for yeasts that depend on mtDNA replication such as *Kluyveromyces lactis* [10], but is dispensable for *S.cerevisiae*.

Mgm101p has been implicated in recombination repair [11]–[13] and the initiation of mtDNA replication [14]. It has been found in association with the Mmm1 protein [15], that is required for maintenance of mitochondrial shape [16]. New data indicates that Mmm1p is part of a complex that attaches the endoplasmic reticulum to the outer mitochondrial membrane [17]. However, Mmm1p also appears to associate with Mgm101p in a structure spanning inner and outer mitochondrial membranes that persists in mutants of yeast that lack mtDNA (rho-zero cells). In other words Mgm101p and Mmm1p do not depend on mtDNA as a scaffold for assembly.

In *S.cerevisiae* it has been shown that the carboxy-terminal two-thirds of the protein, termed the functional or active core of 165 amino acids, can restore growth at 35°C of a temperature sensitive mutant [9]. However, the functional core is unable to complement a *mgm101* null mutant indicating that for proper operation the active enzyme must be a dimer or multimer with input from the amino-terminus of the full-length protein. As the functional core of Mgm101p contains a large number of lysine and arginine residues it is reasonable to believe that this region is responsible for DNA binding and consequent activities. However, the role of the essential amino-terminus remains unknown.

The *MGM101* gene is widely distributed in fungi, some protists and cnidaria but it is not present in plants or the Bilateria. Alignment of amino acids from Mgm101p shows a high level of conservation in the carboxy-terminus [9], whereas a smaller amino-terminal segment is variable in both length and sequence. In view of these observations it became apparent that this protein has two distinct domains. Recent knowledge shows that some proteins are intrinsically disordered or have disordered domains [18]–[20]. Consequently, we were curious to know if the amino-terminus of Mgm101p could belong to the latter category, and if so, whether such a trait could have functional significance.

Intrinsically unstructured/disordered proteins (IDPs) do not adopt a well-defined structure in isolation, instead existing as a rapidly interchanging ensemble of conformations [18]–[20]. The function of disordered proteins relies on this highly flexible state, defying the traditional structure-function paradigm [21]. IDPs participate in many vital cellular functions, including regulation, transcription, translation and signal transduction [22]. They are often involved in binding to other proteins, DNA or RNA and can facilitate the assembly of large multiprotein complexes [23]. The importance of protein disorder is underlined by the abundance of partially or fully disordered proteins in available genome sequences [24], [25]. Correlating with the complexity of the organism, prokaryotic proteins in general display a low amount of structural disorder while eukaryotes have a significantly higher fraction of disordered proteins. Disordered regions can be predicted from the amino acid sequence [26]. Dedicated prediction methods use either machine-learning approaches or simple biophysical models to discriminate disordered regions from ordered ones based on their distinct amino acid composition, the increased content of low complexity segments and their different tendency to form regular secondary structure elements [27], [28]. Disordered proteins are also different in terms of their evolutionary behaviour [29]. In most cases, they are less conserved, but the disorder tendency can be maintained without apparent sequence conservation [30]. In general, protein disorder seems to be a crucial invention in evolution that is especially important in larger multi-domain proteins in eukaryotes [31].

As described in this communication, it appears that the experimentally determined functional core of Mgm101p in *S.cerevisiae* corresponds to an ordered domain that is preceded by an amino-terminal disordered region. To examine whether these two domains have specific or general functions we made chimeric proteins from *S.cerevisiae* and the coral *Acropora millepora* by swapping the two regions. By an *in vivo* assay in *S.cerevisiae* we find that the ordered domain of *A.millepora* can functionally replace the yeast core region but the disordered region of the coral protein cannot substitute for its yeast counterpart. In other words, operation of the disordered domain appears to be specific whereas activity of the ordered region is general. An implication from this result is that the disordered region functions by specific interaction with a component of the nucleoid whereas the core region is not so constrained.

## Results

### Ordered and disordered regions in Mgm101p

In a previous publication [9] we experimentally determined that 165 amino acids in the C terminal region of *S.cerevisiae* Mgm101p are necessary for complementation of a temperature sensitive mutation, *Mgm101-1*. We termed this sequence a ‘functional core’ as it is preceded and followed by segments that are not needed for restoration. In the present report we use ‘core’ in a more general sense to include the 6 inessential carboxy-terminal amino acids. As the functional core region of *A.millepora* Mgm101p has not been determined, we use the more general terminology of ‘core’ in the first instance (see below), to include all amino acids downstream of the junction with the disordered domain.

**Figure 1:**
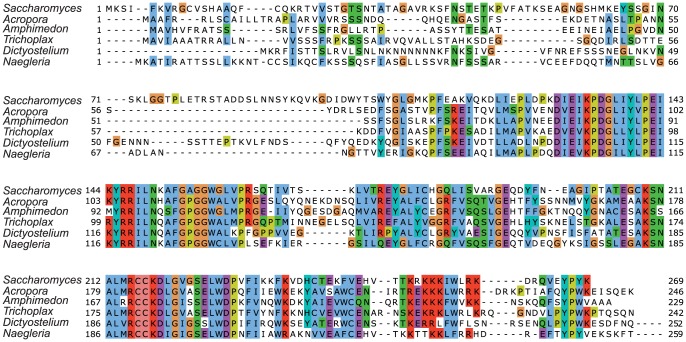
Alignment of six representative members of the Mgm101p sequence family. The C-terminal extension for *Dictyostelium discoideum* and *Naegleria gruberi*, that lack sequence conservation, were omitted from the alignment.

**Figure 2:**
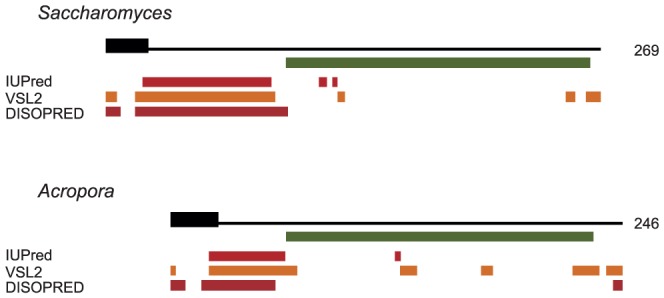
Domain organization for the *S.cerevisiae* and *A.millepora* Mgm101p sequences. The thick black line represents the predicted mitochondrial target signal. The green bar identifies the experimentally determined core region for *S*. *cerevisiae* and the corresponding region determined from the sequence alignment for *A. millepora.* The orange bars represent predicted disordered regions. Disorder predictions were carried our using three independent methods, IUPred, PONDR VSL2, and DISOPRED2. The sequences were aligned so that the beginning of the core region is in the same vertical position.

Using various bioinformatic tools, the sequences of Mgm101p from *S.cerevisiae* and *A.millepora* were studied from the viewpoint of protein disorder, domain content and mitochondrial target sequence. The alignment of the sequences confirmed earlier results, indicating a strong sequence conservation within the C-terminal region corresponding to the functional core, and the lack of apparent sequence conservation within the N-terminal regions (Fig. 1). For both the *S.cerevisiae* and *A.millepora* sequences, the N-terminal region contains a predicted mitochondrial targeting sequence followed by regions that were consistently predicted to contain a large disordered segment using various disorder prediction tools (Fig. 2). A few disordered residues were also predicted within the functional core and in the C-terminal regions. These are likely to correspond to flexible regions within an ordered domain. For both *S.cerevisiae* and *A.millepora*, the analysed sequence features indicated three distinct regions. These correspond to the mitochondrial signal sequence that is cleaved from the mature protein, a disordered region and the C-terminal Mgm101p domain corresponding to the functional core. For *S.cerevisiae* Mgm101p, the lengths of these regions were determined to comprise 29, 68, and 172 residues, respectively. In the case of *A.millepora*, the corresponding regions were 28, 31, and 187 residues.

An extended analysis of the Mgm101 family included four additional sequences from *Amphimedon queenslandica* (sponge), *Trichoplax adhaerens* (placozoan), *Dictyostelium discoideum* (slime mould) and *Naegleria gruberi* (amoebo-flagellate). These species were selected to represent the evolutionary diversity within this family. Their Mgm101p sequences show a very similar domain organization to *S.cerevisiae* and *A.millepora* (Fig. S1). The N-terminal region contains the predicted mitochondrial signal sequence followed by a largely disordered region. These disordered regions lack sequence conservation and also vary in their lengths. The C-terminal region contains the conserved Mgm101 domain, the functional core of the protein. The sequences of *D.discoideum* and *N.gruberi* have a C-terminal extension that lacks sequence conservation and is predicted to be largely disordered (Fig. S1). An even larger C-terminal extension has been described in a Mgm101-like protein from *Physarum polycephalum* termed Glom2, as it participates in DNA agglomeration [32]. Like the two C-terminally extended proteins the *P.polycephalum* carboxy-terminal region lacks sequence conservation and is predicted to be disordered (unpublished data). Apart from these three cases, the domain organization is common to all members of this sequence family, despite the fact that sequence and length of the disordered regions are not conserved. The lack of conservation in the N-terminal region suggests that the mitochondrial targeting signal and the disordered region operate in a species-specific manner.

### Complementation of the temperature sensitive mutation

**Figure 3:**
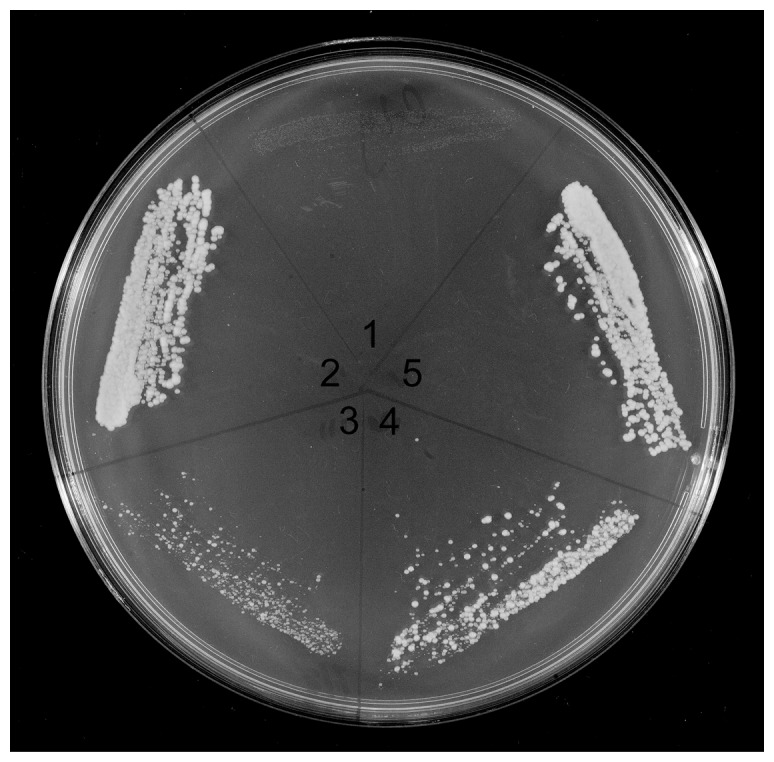
Complementation of the temperature sensitive mutant. A GlyYP plate with 1, M2915-7C *mgm101-1*ts, and M2915-7C transformed with pCXJ22 plasmids containing, 2, *S.cerevisiae MGM101*, 3, *A.millepora MGM101* with a *S.cerevisiae* mitochondrial targeting signal (A.m.ID-A.m.C)(Fig. 3), 4, *S.cerevisiae* intrinsically disordered (ID) domain joined to *A.millepora* core region (S.c.ID-A.m.C) and 5, *A.millepora* ID region joined to *S.cerevisiae* core region (A.m.ID-S.c.C). The constructs all have a mitochondrial targeting signal sequence as shown in Figure S2. The plate was incubated at 35°C for 3 days before being photographed.

In a previous study we used a recessive temperature-sensitive mutant, *mgm101-1* P141S, located at the end of the first highly conserved region (Fig. 1), to determine that Mgm101p has a 165 amino acid functional core [9]. The same assay was used in the present work to examine whether the *A.millepora* Mgm101p and two chimeric proteins can restore growth of the mutant at the restrictive temperature. Three plasmids, one containing the *MGM101* gene from *A.millepora*, and two containing chimeric genes having the intrinsically disordered domain (ID) of yeast Mgm101p joined to a putative core region of the coral gene and vice versa, were constructed in the vector pCXJ22 used in the previous study [9] (Fig.S2). These plasmids, together with the original pCXJ22-ScMGM101, all have the mitochondrial targeting signal sequence specific for *S.cerevisiae*. The strain M2915-7C, containing the *mgm101-1* temperature sensitive (ts) mutation was transformed with the three plasmids followed by selection for Ura^+^. Transformants were examined for restoration of growth at the restrictive temperature, 35°C (Fig. 3). All three strains, as well as the strain with the wild-type *S.cerevisiae* gene, grow at the restrictive temperature. Of note is that the growth rate at 35°C varies. The transformant containing pCXJ22-AmMGM101 (A.m.ID-A.m.C) grows slower than the transformant containing the ID region of *S.cerevisiae* joined to the coral core domain (S.c.ID-A.m.C). Almost equal growth rates are displayed by the transformants containing the wild-type yeast gene and the ID region of the coral linked to the *S.cerevisiae* core domain (A.m.ID-S.c.C).

### Restoration of respiration with a chimeric protein

**Figure 4:**
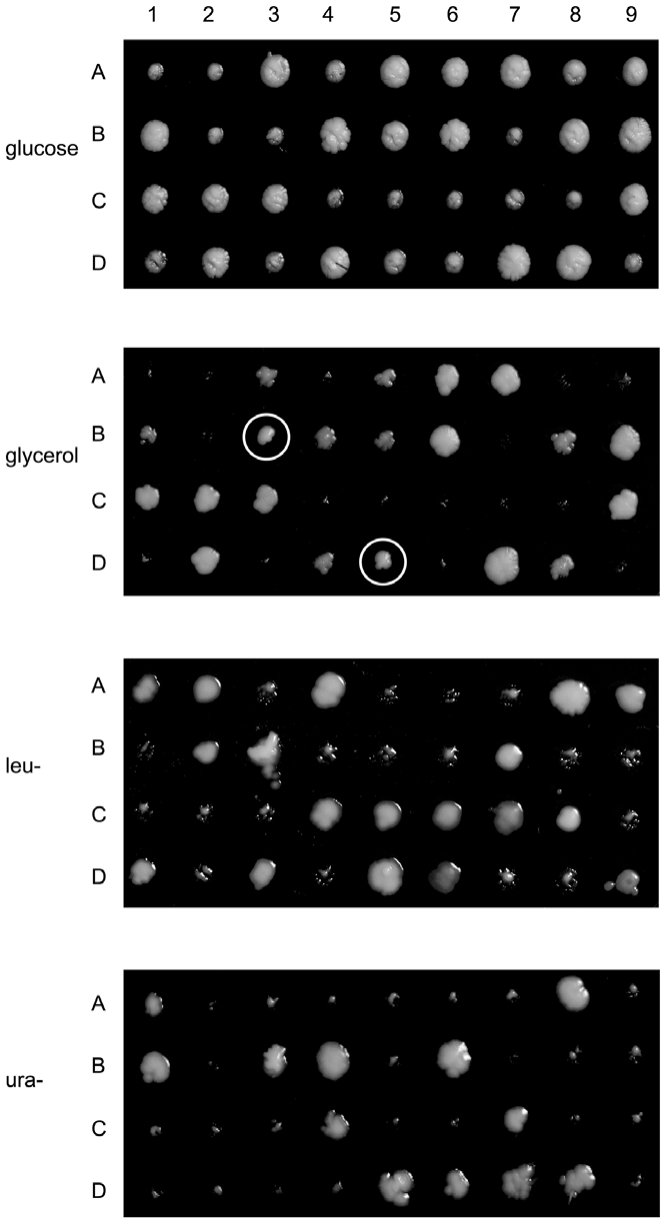
Maintenance of respiration by the core of *A.millepora* Mgm101p. Tetrads of *S.cerevisiae* CS5/mL3 containing pCXJ22- *S.cerevisiae* ID domain-*A.millepora* core region. After growth on GYP for 35 hours at 30°C phenotypes were determined by replica plating to GlyYP, GMM supplemented with Ade,His,Leu (Ura^+^ determination), and GMM supplemented with Ade,His,Ura, (Leu^+^ determination). Plates were incubated at 30°C for 3 days before being photographed. The white circle identifies colonies 3B and 5D that grow on all media.

The above result led us to test whether restoration of respiration could occur in the absence of the Mgm101-1ts mutant protein. For this test we used a diploid strain, CS5/mL3, homozygous for *ura3* and heterozygous for disruption of *MGM101* (*mgm101*::*LEU2*). The same pCXJ22 constructs, used before, were employed for transformation of the diploid. Transformants were sporulated, asci dissected and results from the four strains are summarised in Table 1. The presence of the plasmid, marked by Ura^+^ (the plasmid contains URA3 wild type) [9] varied between 54–62% while disruption of *MGM101*, indicated by Leu^+^ (disruption of *MGM101* is by insertion of *LEU2* wild type), segregates 2∶2. A demonstration that wild-type *ScMGM101* can complement disruption of the chromosomal gene is shown in Table 1. All 63 Ura^+^,Leu^+^ spores can grow on glycerol. Growth on glycerol is an indication that respiration is present that in turn depends on a functional mitochondrial genome and an operational Mgm101p. By contrast, no complementation was found with the *A.millepora* gene or when the ID region of this gene is attached to the core region of *S.cerevisiae* mgm101p (A.m.ID-S.c.C). However, when the ID domain of the yeast gene is joined to the core region of the coral gene (S.c.ID-A.m.C), 10 out of 42 Ura^+^ Leu^+^ grow on glycerol (Fig. 4). As shown in the figure, there are two tetrads (3&5) with three colonies growing on glycerol. In each of these tetrads there is one Gly ^+^ colony, 3B and 5D, that contains both the plasmid (Ura^+^) and disruption of *MGM101* (Leu^+^).

**Table 1:** Distribution of phenotypes in segregants containing pCXJ22 *MGM101* plasmids.

Plasmid Construct	Asci with 4 viable spores	Ratio of Gly^+^: Gly^−^ spores*			Ura^+^ spores, frequency(%)	Ura^+^, Leu^+^ spores	
		4∶0	3∶1	2∶2		Gly^+^	Gly^−^
*S.c MGM101*	56	19	24	13	139 (62%)	63	0
*A.m MGM101*	34	0	0	34	74 (54%)	0	31
*S.c* 1D domain-*A.m* core*	44	2	6	35	98 (56%)	10	32
*A.m* 1D domain-*S.c* core	50	0	0	50	108 (54%)	0	44

* 1 tetrad contains 4 Gly^−^ spores.

Examination by DAPI staining of Ura^+^,Leu^+^ cultures that failed to grow on glycerol revealed that mtDNA was no longer present. A possible explanation for failure to maintain mtDNA is that the chimeric protein has lower activity compared to the wild type and only in some cases is there sufficient plasmid to permit the remaining activity to function productively. Support for this view is that the expression of the MGM101 constructs relies on the relatively weak native promoter and that pCXJ22 depends on the ARS.CEN sequence for replication and is in low copy number as demonstrated by the limited frequency of Ura ^+^ (54–62%) in tetrads. This is also illustrated in Figure 4 by the absence of Ura^+^ from some tetrads (2&9).

### Examination of constructs using an integrative plasmid

In view of the variable presence of pCXJ22 in tetrad colonies, we hypothesized that integration of the constructs into a chromosomal location would improve frequency of growth on glycerol, especially of transformants containing the ID domain of the yeast gene joined to the coral core of Mgm101p. For this test we recloned the three constructs into the integrative vector pUC-n lacking an intrinsic origin of DNA replication. Such constructs can be integrated at *ura3* by cleavage at a single *Stu*I site in the wild-type *URA3* gene on the plasmid followed by selection for Ura^+^ transformants. All resulting isolates had a stable genotype that segregated 2∶2 for *URA3:ura3* on tetrad dissection.

When *S.cerevisiae MGM101* was integrated, all spores containing a Ura^+^,Leu^+^ phenotype grew on glycerol (Table S1). However, none of the other constructs showed greater than 2∶2 segregation of Gly^+^∶Gly^−^ growth or contained any Ura^+^,Leu^+^ spores that were Gly^+^. It is likely, as with pCXJ22, that the wild-type *MGM101* promoter is not active enough to yield sufficient protein to maintain mtDNA except when driving expression of the native *S.cerevisiae* protein.

### Function of the intrinsically disordered domain

**Figure 5:**
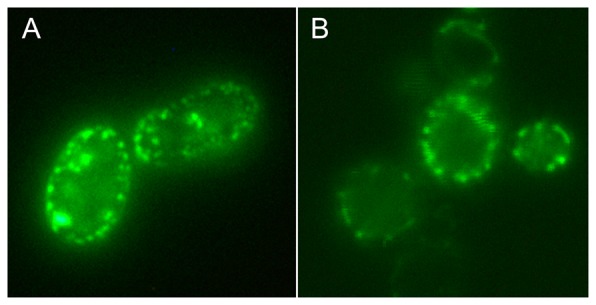
Nucleoids labelled with GFP. *S. cerevisiae* M2915-7C transformed with (A) pCXJ8MGM101-GFP and (B) the deletion plasmid pCXJ8MGM101Δ99-269-GFP subcultured to GMM Ade,His,Leu for 24 h at 30°C before being photographed. The deletion leaves 98 amino acids at the N-terminus of Mgm101p consisting of a 22 amino acid mitochondrial targeting signal and 76 amino acids incorporating the ID region.

Previous studies employing green fluorescent protein (GFP) have shown that Mgm101p is found in mitochondrial nucleoids [12], [15]. Similar observations were obtained in our laboratory with a centromeric vector, pCXJ8, containing GFP joined to the C-terminus of Mgm101p (Xiaoming Zuo, unpublished observations). In the present study we have used the pCXJ8-MGM101GFP plasmid (Fig. S4) as a starting point to construct a plasmid with the N-terminal ID region of Mgm101p joined to GFP. This plasmid has the 22 amino acid mitochondrial targeting signal and the next 76 amino acids containing the ID region linked to GFP. Using this construct we find that GFP has a punctate appearance and a peripheral distribution in cells that is similar to that obtained with the plasmid containing full-length Mgm101p (Fig. 5).

## Discussion

Prediction by three different methods shows that the previously determined 165 amino acid functional core of *S.cerevisiae* Mgm101p is not disordered whereas the N-terminal region, as well as part of the mitochondrial signal sequence, is disordered. Sequence alignment of Mgm101 proteins shows that the N-terminal domain does not contain recognizable common elements while the functional core shares conserved sequences. We have used the predictions of the ordered and disordered domains of *A.millepora* and the conserved core of *S.cerevisiae* in constructing the chimeric proteins.

Examination of constructs by complementation of the temperature sensitive allele, *Mgm101-1*, has shown that the wild types and chimeras all restore temperature tolerance but at different levels (Fig. 3). The complementation test only determines whether the core region can help restore temperature tolerance. Restoration is likely to depend on a hybrid multi-subunit complex. Recent studies have shown that Mgm101p forms a ring structure containing 14 subunits [11], [13]. Hence a hybrid complex of subunits from different sources may function at the restrictive temperature because unfolding of Mgm101-1p may be prevented by the foreign subunits. In the case of *A.millepora* wild type or the yeast ID-coral core region construct, restoration is not as effective as the *S.cerevisiae* wild type or coral ID-yeast core region construct (Fig. 3). However, complementation with *A.millepora* wild type or the yeast ID-coral core region chimeric construct does suggest that the coral core domain may function. However, complementation does not demonstrate that the *A.millepora* core region of Mgm101p can operate in mtDNA maintenance in the absence of a contribution from the yeast Mgm101-1 ts protein.

Evidence that the core region of *A.millepora* Mgm101p can function in maintaining respiration in the absence of the Mgm101-1 protein has been obtained by a genetic test that simultaneously gives prominence to the ID region of yeast Mgm101p. It has been found that the core-region of the coral protein can maintain respiration when attached to the yeast ID region. However, respiration is not maintained when the yeast core-region is joined to the coral ID domain despite the demonstrated integrity of the yeast core (Fig. 3). In other words, the ID region of the yeast protein is vital for the activity of the coral core region. In summary, the two constructs with the coral ID region of Mgm101p do not function whereas the construct with the yeast ID domain does.

Previous studies have shown that isolated nucleoids, both from *S.cerevisiae* [33] and *Candida parapsilosis* [5], contain Mgm101p while the Mgm101-like protein Glom2 is also found in nucleoids [32]. An *in vivo* demonstration of Mgm101p's association with nucleoids has been obtained using GFP [12], [15]. Consequently we considered whether the association of Mgm101p with the nucleoid may be due to the ID region. In the present study we found that the ID domain of Mgm101p is sufficient to give a punctate appearance of GFP in the absence of the core region (Fig. 5). The distribution of GFP at the periphery is characteristic of nucleoids being located in mitochondria that are close to the cell wall. In a comprehensive study where proteins, or fragments of proteins, from different compartments of mitochondria were labelled with GFP, only Abf2, that binds to mtDNA, gave a punctate appearance whereas mitochondrial tubule fluorescence was found with other components [34]. Therefore it appears that the ID region of Mgm101p can direct GFP to the nucleoid.

In our study we have shown that Mgm101p is a bifunctional protein with separate activities associated with its two domains. The specific function of the N-terminal region is likely to depend on its disordered nature. Disordered segments are frequently involved in the assembly of large macromolecular complexes [35]. In accord with the idea that ID domains are malleable, it seems possible that this portion of Mgm101p could mould to a structural template in the nucleoid where operation of its core would be undertaken. Future work will attempt to identify the interacting partners of Mgm101p and further define the roles of the two domains. If the ID domain of Mgm101p is confirmed as necessary, as well as sufficient, for nucleoid localization, it is expected that the coral ID domain will be unable to provide this function, possibly due to an inability to recognize the interacting molecules in yeast.

The bifunctional nature of Mgm101p underlines the importance of ID regions even in mitochondria where the amount of protein disorder is generally low [36].

## Materials and Methods

### Strains and media

The yeast strains are M2915-7C *Mat*a,*ade2-1,his4,leu2-3,ura3-52,mgm101-1*ts, and CS5/mL3 *Mat*a/*Mat*α,*leu2-3/leu2-3,ura3-52/ura3-52,ade2-1/+,his4/+, mgm101::LEU2/+.* GYP medium contains 1% glucose, 0.5% Bacto yeast extract, 1% Bacto tryptone; GlyYP contains 2% glycerol in place of glucose and GMM; glucose minimal medium contains, 1% glucose, 0.5% ammonium sulfate and 0.67% Difco yeast nitrogen base without amino acids. Nutrients essential for auxotrophic strains were added at 25 µg/ml for bases and 50 µg/ml for amino acids. Sporulation medium consists of 1% potassium acetate and 0.05% glucose. Plates were solidified by 2% Difco agar.

### Biological material

Staged coral larvae and adult material was collected during the annual mass spawning event at Magnetic Island, Queensland, Australia under permit G08/28473.1 issued by the Great Barrier Reef Marine park Authority.

### Sequence analysis

The alignment was generated using the Clustalw [37] and Jalview [38] programs. The presence of a mitochondrial target signal was predicted using the MitoProt program [39]. Protein disorder was predicted using three largely orthogonal methods, the IUPred [40], Disopred2 [25], and PONDR VSL2 [41] programs. For Disorpred2 and PONDR VSL2 the default cutoff value was used, for IUPred it was lowered to 0.4 to allow regions undergoing disorder-to-order transitions to be predicted as disordered. Currently, disorder prediction methods work with about 80% accuracy overall but can be quite noisy [27]. The main reason for this is that protein disorder is a heterogeneous phenomenon and the various methods can recognize the different types of disorder differently. For this reason, it is not clear which is currently the best method or what is the best way to create a consensus. Nevertheless, the agreement of the prediction methods confirms that our conclusions are not dependent on the choice of prediction method.

### Isolation of an MGM101 cDNA clone from *A.millepora*

Total RNA was isolated from frozen coral tissue ground in liquid nitrogen using the RNAwiz reagent (Ambion) following manufacturer's instructions. cDNA was synthesized from mixed planula larva and adult RNA using an anchored oligo dT primer and PrimeScript reverse transcriptase (Takara). PCR primers based on the MGM101 sequence in the *A.millepora* transcriptome database [42] (Accession JR994989) were used to amplify a MGM101 product, which was cloned into pGEMTeasy (Promega, Madison, WI). The primers were 5′ GATCTTCTACTAGTGATCAACAACATCAAGAAAATGG 3′ and 5′ GGCCACATGAATTCTCTTTCTCACACTGGATGGCAAG 3′. These primers contain *Spe*I and *Eco*RI restriction endonuclease sites to facilitate cloning. The plasmid insert was sequenced with internal and vector primers using Big Dye Terminator v. 3.1 (Applied BioSystems) and reactions were run on an ABI 3730 DNA Analyzer at the Biomolecular Resource Facility (JCSMR, ANU).

### Constructs for yeast transformation

The *A.millepora MGM101* cDNA insert was excised with SpeI and EcoRI and inserted between the *Spe*I and *Eco*RI sites of pCXJ22-ScMGM101 [9] to give pCXJ22-AmMGM101 which codes for a protein consisting of the *A.millepora* intrinsically disordered region and Mgm101p core downstream of the *S.cerevisiae* mitochondrial import signal sequence. To make a construct with an ORF consisting of the *S.cerevisiae* intrinsically disordered amino terminal region fused to the *A.millepora* Mgm101p core the primers, 5′ GTTAGCGGAATTCACTGGCCGTCGTTTTACAACGTC 3′ and 5′ CCAGTCGGTACCTCCCTTGACTTGCTTATAACTATTG 3′ were used to amplify a product from pCXJ22-ScMGM101, and the primers 5′ TCATATGGTACCCTATCAGAAGACTTTTCTGGAGCTTC 3′ and 5′ GGCCACATGAATTCTCTTTCTCACACTGGATGGCAAG 3′ were used to amplify a product from the *A.millepora MGM101* cDNA clone. The products were digested with *Acc*651 and *Eco*RI and ligated together to give pScAmMGM101. To make a construct with an ORF consisting of the *A.millepora* intrinsically disordered amino terminal region fused to the *S.cerevisiae* Mgm101p core the primers, 5′ GTTAGCGGAATTCACTGGCCGTCGTTTTACAACGTC 3′ and 5′ CCAGAAAAGGTACCTGATAGCCTATCATATGAATTATTCG 3′ were used to amplify a product from pCXJ22-AmMGM101 and primers 5′ GACTGGGGTACCTCATGGTATGGCCTAGGTATGAAGC 3′ and 5′ GGCCAGTGAATTCCGCTAACCCTGAAATAGAAGGCG 3′ were used to amplify a product from pCXJ22-ScMGM101. The products were digested with *Acc*651 and *Eco*RI and ligated together to give pAmScMGM101. The amino acid sequences of the Mgm101 proteins coded by these constructs are shown in Figure S3.

To make the integrative constructs, fragments containing the intrinsically disordered and core domains were excised from pCXJ22-AmMGM101, pScAmMGM101, and pAmScMGM101 with *Spe*I and *Eco*RI and ligated into the *Spe*I and *Eco*RI sites of pUC-n-MGM101His [9] [43].

To make a construct containing the *S.cerevisiae* Mgm101p intrinsically disordered region fused to GFP in a pCXJ8 vector (placing expression of the fusion under the control of the alcohol dehydrogenase promoter), an *Eco*RI fragment containing the *S.cerevisiae MGM101* open reading frame fused to GFP was excised from pCXJ8-MGM101GFP [43]; Fig. S4), and inserted into the *Eco*RI site of pEMBL8+ [44] to give pEMBLMGM101GFP. A region corresponding to amino acid position 99 to the C terminus of the Mgm101 protein was deleted from pEMBLMGM101GFP using *Pfu* Ultra II Fusion HS DNA polymerase (Agilent Technologies) with a method modified from that described in [45]. The primers pairs used were 5′ CAAGTCAAGGGAGACAGTAAAGGAGAAGAACTTTTCAC 3′; 5′ TTATAACTATTGTTCAAAGAATC 3′ and 5′ TTCTTCTCCTTTACTGTCTCCCTTGACTTGCTTATAAC 3′; 5′ CTTTTCACTGGAGTTGTCCC 3′. PCR reactions were digested with *Dpn*I (New England Biolabs) to remove template DNA and purified with the QIAquick PCR Purification Kit (Qiagen). 50 ng of each PCR product were combined in 10 mM Tris pH 7.5, 100 mM NaCl, 1 mM EDTA heated to 95°C for 3 minutes and annealed at 65°C for 2 minutes followed by 15 minutes at 25°C. Colonies harbouring the desired deletion construct were recovered following transformation of JM109 (Promega) with the annealed products. The resulting plasmid, pEMBLMGM101Δ99-269GFP, was digested with *Eco*RI and the purified insert was ligated into the *Eco*RI site of pCXJ8 to give pCXJ8-MGM101Δ99-269GFP. The domain structure of the wild-types and chimeric constructs is illustrated in Figure S2, while amino acid sequences of these proteins are shown in Figure S3.

### Manipulation of *S.cerevisiae*

Transformation of yeast was performed by the lithium acetate-dimethyl sulfoxide method [46]. Tetrad dissection employed a Singer 200 series micro-dissection apparatus (Singer Instruments, Somerset, UK) following a brief treatment with Zymolyase (Seikaguku, Japan).

### Microscopy

Digital images of yeast cells were captured with a Spot Camera mounted on a Leica DM6000B microscope with 63× or 100× NA1.4 oil immersion objectives.

## Supporting Information

Figure S1 Domain organization for the six representative members of Mgm101p sequence family. The thick black line represents the predicted mitochondrial target signal. The green bar indicates the experimentally determined core region for *S*.*cerevisiae* and the corresponding region determined from the sequence alignment for *A.millepora.* The orange bars represent predicted disordered regions. Disorder predictions were carried out using three independent methods, IUPred, PONDR VSL2, and DISOPRED2, indicated by different shades. The sequences were aligned so that the beginning of the core region is in the same vertical position.
(EPS)

Figure S2 Structure of the Mgm101p constructs. The sequence regions for Mgm101p, indicating the mitochondrial target sequence (S.c.T/A.m.T) in blue, the disordered region (S.c.ID/A.m.ID) in red and orange respectively, and the core region (S.c.C/A.m.C) in green for *S.cerevisiae* and *A.millepora*. Based on these regions three constructs were designed. Construct 1 (A.m.ID-A.m.C) has the *A.millepora* disordered and *A.millepora* core regions fused to the *S.cerevisiae* mitochondrial import signal. Construct 2 (S.c.ID-A.m.C) has the *S.cerevisiae* mitochondrial import signal and disordered region fused to the *A.millepora* core. Construct 3 (A.m.ID-S.c.C) has the *A.millepora* disordered region and the *S.cerevisiae* core fused to the *S.cerevisiae* mitochondrial import signal. In the last two cases the dipeptide GlyThr was introduced by the restriction site (*Acc*651) that was used to facilitate the cloning.
(EPS)

Figure S3 Amino acid sequences of the construct open reading frames. AmMgm101p. The predicted sequence of the *A.millepora* MGM101 protein derived from Accession JR994989 and the *A.millepora* genome sequence (www.coralbase.org). ScMgm101p. The sequence of the *S.cerevisiae* MGM101 protein (Accession NP_012678) A.m.ID-A.m.C. (pCXJ22-AmMGM101). This construct consists of the *S.cerevisiae* mitochondrial targeting signal and the *A.millepora* ID and core regions. S.c.ID-A.m.C. (pScAmMGM101). This construct consists of the *S.cerevisiae* mitochondrial targeting signal and ID region and the *A.millepora* core region. A.m.ID-S.c.C. (pAmScMGM101). This construct consists of the *S.cerevisiae* mitochondrial targeting signal, the *A.millepora* ID region and the *S.cerevisiae* core. S.c.MGM101GFP. The sequence of the MGM101-GFP fusion protein from pCXJ8-MGM101GFP. S.c.ID-GFP. (pCXJ8MGM101Δ99-269GFP). The sequence of the *S.cerevisiae* ID region fused to GFP. It represents a deletion of amino acids 99–272 from S.c.MGM101GFP (A three amino acid linker between the end of the Mgm101 protein and GFP (Fig. S3), which is present in S.c.MGM101GFP, has been deleted in S.c.ID-GFP along with the core region).
(TIF)

Figure S4 Map of plasmid pCXJ8-MGM101GFP. This plasmid contains the full-length *S.cerevisiae* MGM101 open reading frame fused in-frame to GFP (*Aequorea victoria* GFP-S65T derived from pFA6a-GFPS65T-kanMX6; accession AJ002682). Expression of the fusion protein is under the control of the *S.cerevisiae ADH* (alcohol dehydrogenase) promoter.
(TIF)

Table S1 Distribution of phenotypes in segregants from pUC-N *MGM101* constructs integrated at *ura3*
(DOC)
