# Supplementary figures and images for: The N-Terminal Intrinsically Disordered Domain of Mgm101p Is Localized to the Mitochondrial Nucleoid
Source: PLoS One. 2013 Feb 13;8(2):e56465. doi: 10.1371/journal.pone.0056465 (PMC3572067; doi:10.1371/journal.pone.0056465)

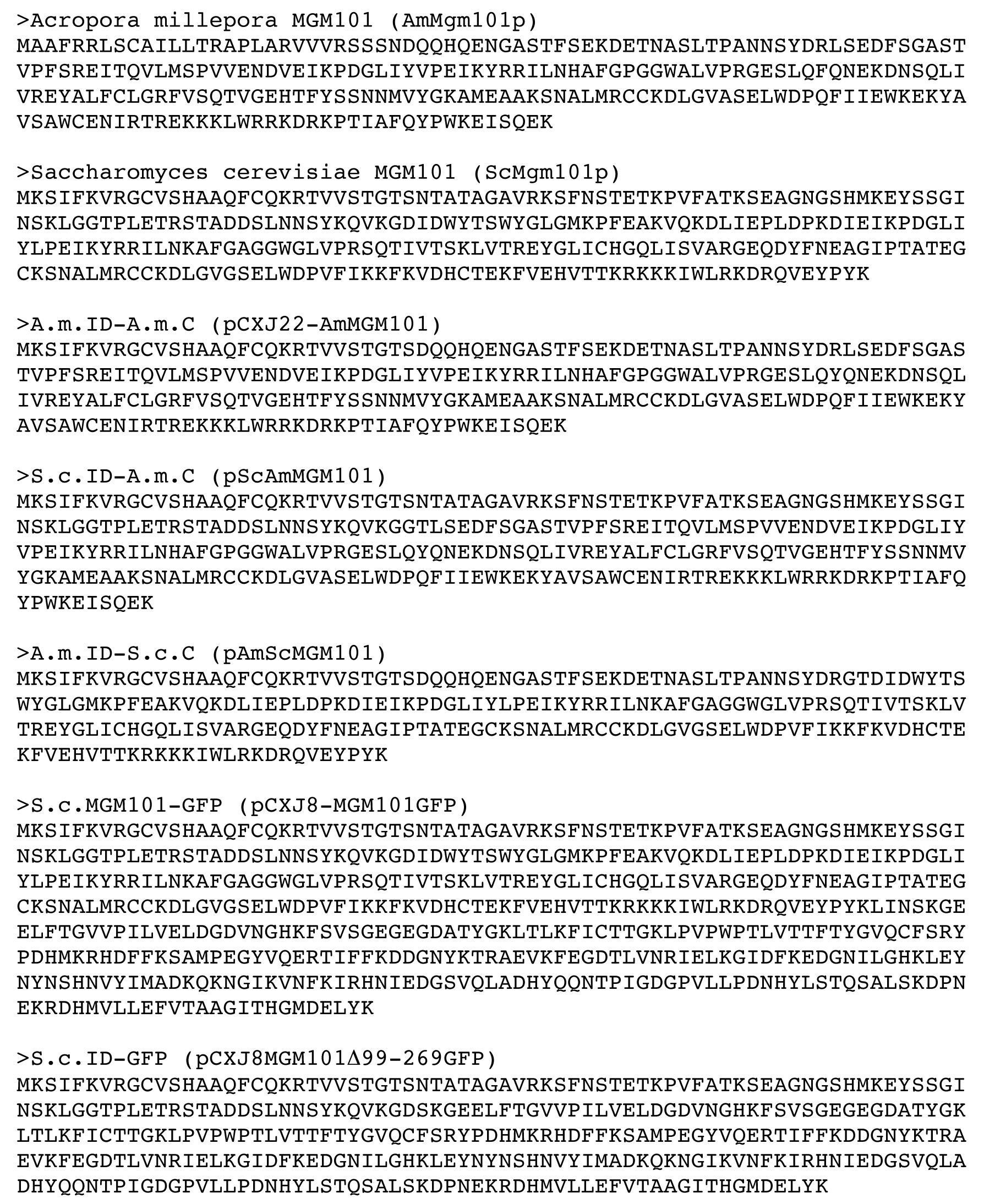

Supplement: Figure S3 — Amino acid sequences of the construct open reading frames. AmMgm101p. The predicted sequence of the A.millepora MGM101 protein derived from Accession JR994989 and the A.millepora genome sequence (www.coralbase.org). ScMgm101p. The sequence of the S.cerevisiae MGM101 protein (Accession NP_012678) A.m.ID-A.m.C. (pCXJ22-AmMGM101). This construct consists of the S.cerevisiae mitochondrial targeting signal and the A.millepora ID and core regions. S.c.ID-A.m.C. (pScAmMGM101). This construct consists of the S.cerevisiae mitochondrial targeting signal and ID region and the A.millepora core region. A.m.ID-S.c.C. (pAmScMGM101). This construct consists of the S.cerevisiae mitochondrial targeting signal, the A.millepora ID region and the S.cerevisiae core. S.c.MGM101GFP. The sequence of the MGM101-GFP fusion protein from pCXJ8-MGM101GFP. S.c.ID-GFP. (pCXJ8MGM101Δ99-269GFP). The sequence of the S.cerevisiae ID region fused to GFP. It represents a deletion of amino acids 99–272 from S.c.MGM101GFP (A three amino acid linker between the end of the Mgm101 protein and GFP (Fig. S3), which is present in S.c.MGM101GFP, has been deleted in S.c.ID-GFP along with the core region). (TIF) [file pone.0056465.s003.tif]

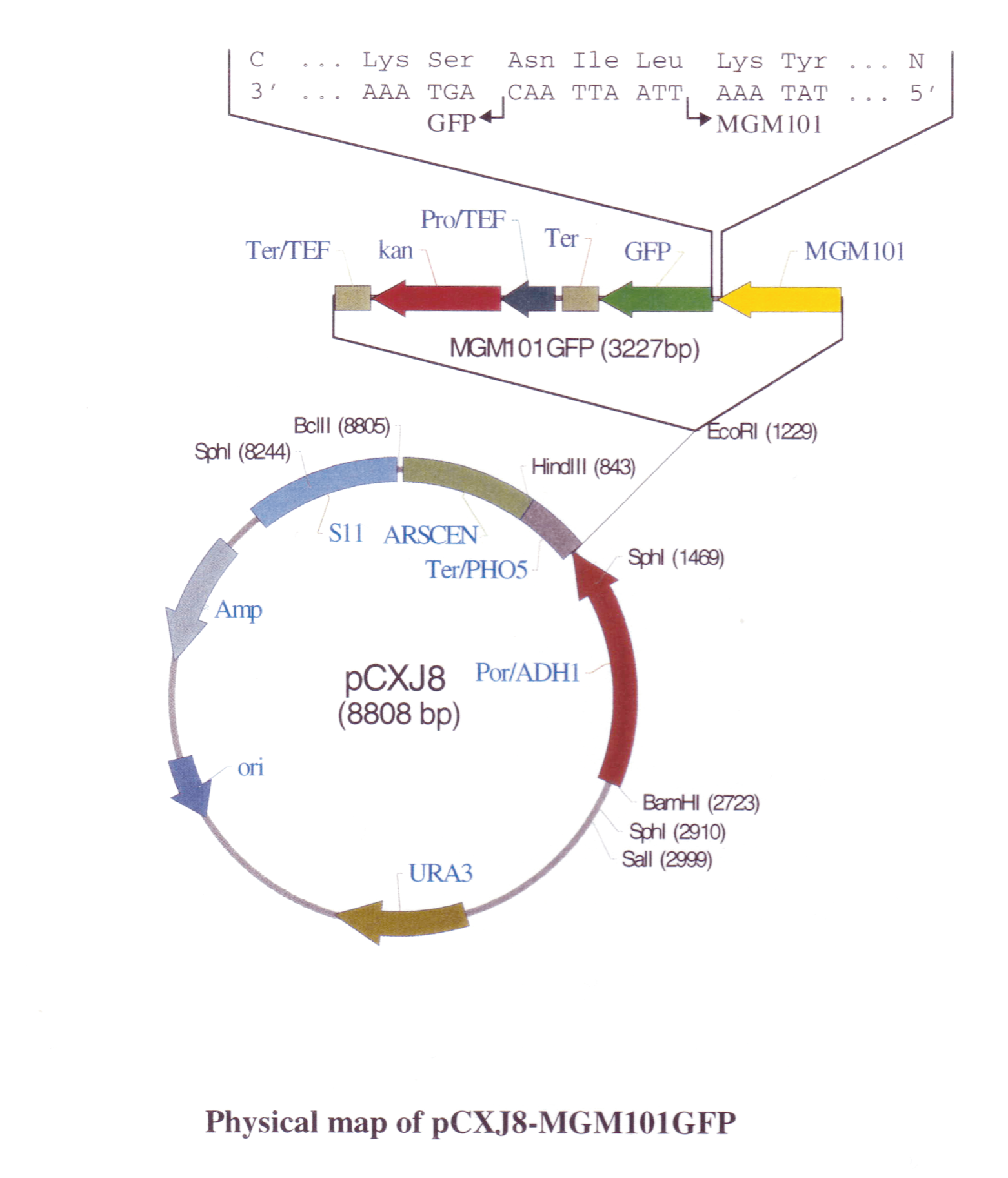

Supplement: Figure S4 — Map of plasmid pCXJ8-MGM101GFP. This plasmid contains the full-length S.cerevisiae MGM101 open reading frame fused in-frame to GFP (Aequorea victoria GFP-S65T derived from pFA6a-GFPS65T-kanMX6; accession AJ002682). Expression of the fusion protein is under the control of the S.cerevisiae ADH (alcohol dehydrogenase) promoter. (TIF) [file pone.0056465.s004.tif]
