# Supplementary material for: The N-Terminal Intrinsically Disordered Domain of Mgm101p Is Localized to the Mitochondrial Nucleoid
Source: PLoS One. 2013 Feb 13;8(2):e56465. doi: 10.1371/journal.pone.0056465 (PMC3572067; doi:10.1371/journal.pone.0056465)
Supplement: Table S1 — Distribution of phenotypes in segregants from pUC-N MGM101 constructs integrated at ura3 (DOC) [file pone.0056465.s005.doc]

Table S1. Distribution of phenotypes in segregants from pUC-N *MGM101* constructs integrated at *ura3*

| **pUC-N-Construct** | **Asci with 4 viable spores** | **Ratio of Gly^+^: Gly^-^ spores*** | | | **Ura^+^, Leu^+^ spores** | |
| --- | --- | --- | --- | --- | --- | --- |
|  |  | **4:0** | **3:1** | **2:2** | **Gly^+^** | **Gly^-^** |
| *S.c MGM101* | 51 | 11 | 32 | 8 | 54 | 0 |
| *A.m MGM101* | 34 | 0 | 0 | 33 | 0 | 31 |
| *S.c* 1D domain *A.m core* | 33 | 0 | 0 | 32 | 0 | 36 |
| *A.m* 1D domain *S.c core* | 32 | 0 | 0 | 30 | 0 | 33 |

*1 tetrad each from *A.m MGM101* and *A.m* ID domain – *Sc* core contain 3 Gly^-^ spores and 1 tetrad each from *S.c* 1D domain – *A.m* core and *A.m* ID domain – *S.c* core contain 4 Gly^-^ spores
